# Phenotypic and Functional Diversity in Meat-Associated *Pseudomonas* Isolates: Motility, Biofilm Formation, and AI-2 Activity

**DOI:** 10.3390/foods15173044

**Published:** 2026-08-28

**Authors:** Yasemin Şefika Küçükata, Beyza Nur Güç, Hasan Yetim, Banu Metin

**Affiliations:** 1 Department of Food Engineering, Faculty of Engineering and Natural Sciences, Istanbul Sabahattin Zaim University, 34303 Istanbul, Türkiye; yasemin.kucukata@izu.edu.tr (Y.Ş.K.); beyzanurguc@gmail.com (B.N.G.); hasan.yetim@izu.edu.tr (H.Y.); 2 Department of Food Engineering, Faculty of Chemical and Metallurgical Engineering, Yildiz Technical University, 34210 Istanbul, Turkey

**Keywords:** autoinducer-2, biofilm, meat, *Pseudomonas*

## Abstract

*Pseudomonas* species are important spoilage bacteria in aerobically stored chilled meat; however, the occurrence of the interspecies quorum sensing signal autoinducer-2 (AI-2) and its association with biofilm formation and motility in these bacteria remain insufficiently understood. In this study, *Pseudomonas* isolates obtained from spoiled beef steak and minced beef were characterized for biofilm formation, motility traits, and AI-2 activity. *RpoD* sequence analysis of the preselected *Pseudomonas fragi*-related isolates revealed a diverse species composition, with *Pseudomonas bubulae* being more frequently identified than *P. fragi*. This finding highlights the importance of updated taxonomic approaches for species-level identification within this group. Thirty-five isolates that were positive in the Congo red agar assay were evaluated for biofilm formation at 4 °C and 25 °C over 7 days. Mean biofilm formation was significantly higher at 25 °C during early incubation, whereas prolonged incubation resulted in comparable biofilm levels under chilled conditions. AI-2 activity was detected in 25 isolates using a *Vibrio campbellii* bioluminescence assay. Principal component analysis showed that AI-2-like activity and biofilm formation contributed to a similar phenotypic dimension, whereas direct Spearman correlation analysis revealed no significant monotonic relationship between these traits. Overall, these findings highlight the phenotypic versatility of meat-associated *Pseudomonas* spp. and suggest that biofilm formation is likely influenced by multiple strain-specific and environmental factors beyond AI-2-like activity.

## 1. Introduction

Fresh meat spoilage during storage and distribution remains a major concern for the food industry due to its impact on product quality, shelf life, and economic losses [1]. Under aerobic chilled storage conditions, psychrotrophic bacteria, particularly *Pseudomonas* spp., are recognized as dominant spoilers of meat products [2,3,4]. Among these, *Pseudomonas fragi* has traditionally been considered one of the major spoilage-associated species [5]. Meat spoilage caused by these bacteria is typically characterized by off-odors, slime formation, and discoloration, rendering products unacceptable for consumption [6]. A major factor contributing to the persistence and spread of spoilage microorganisms in meat-processing environments is biofilm formation [7]. *Pseudomonas* spp. readily colonize both biotic surfaces, such as meat tissues, and abiotic surfaces, such as processing equipment, thereby facilitating cross-contamination and persistence within the production environment [8].

Quorum sensing (QS) is a communication system based on the production and detection of signaling molecules called autoinducers, enabling coordinated regulation of bacterial behaviors such as virulence, motility, and biofilm formation [9,10]. In Gram-negative bacteria, QS is commonly mediated by N-acyl-L-homoserine lactones (AHLs), while Gram-positive bacteria primarily use oligopeptide signals [11,12]. In addition to these systems, autoinducer-2 (AI-2) functions as an interspecies signaling molecule produced by both Gram-positive and Gram-negative bacteria [13]. These signal molecules are involved in regulating biofilm formation and other collective bacterial behaviors in diverse microbial communities [14,15].

*Pseudomonas* species are known to produce and/or respond to different quorum sensing signal molecules, including AHLs, the *Pseudomonas*-specific quinolone signal (PQS), and AI-2 [16,17]. Several *Pseudomonas* species have been reported to produce AHLs. For instance, AHL production has been identified in *P. fragi* [18], *P. psychrophila* [19], *P. putida* [20], *P. fluorescens* [21,22], and *P. aeruginosa* [17]. In addition to AHL-mediated signaling, AI-2 activity has also been reported in certain *Pseudomonas* species, such as *P. fluorescens* [23] and *P. lundensis* [24]. AI-2 activity has only been reported in a limited number of *P. fragi* isolates from spoiled meat [25]. Despite these reports, the occurrence of AI-2 and its relationship with biofilm formation and motility in meat-associated pseudomonads remain poorly understood. This highlights the need for further investigation of AI-2 activity and associated phenotypes in meat-derived *Pseudomonas* isolates.

This study aimed to isolate and identify meat-associated *Pseudomonas* spp. from retail beef steak and minced beef following the development of visible/sensory spoilage signs during aerobic storage at 4 °C, and to characterize their biofilm-forming ability, motility traits, and AI-2 activity, thereby exploring phenotypic relationships among the recovered isolates in the context of chilled meat spoilage.

## 2. Materials and Methods

### 2.1. Pseudomonas Isolation

Beef steak (*n* = 10) and minced beef (*n* = 10) samples were collected from various local meat markets and butcher shops in Istanbul in May 2022. The samples were then incubated aerobically at 4 °C for 7 days (intact pieces) and 5 days (minced) until clear signs of spoilage, such as off-odor and/or discoloration, were observed.

Following spoilage development, meat samples (25 g) were homogenized in 225 mL of Ringer’s solution (Merck, Darmstadt, Germany), and serial dilutions were prepared. Aliquots were then plated onto *Pseudomonas*-specific agar supplemented with CFC (Cetrimide, Fusidate, and Cephloride) (Neogen^TM^, Lansing, MI, USA) and incubated at 25 °C for 24 h. Colonies were selected, replated, and stored at −80 °C in nutrient broth (Merck, Darmstadt, Germany) supplemented with 20% glycerol for further use.

### 2.2. Bacterial Strains

For the AI-2 bioassay, *Vibrio campbellii* ATCC BAA-1117 was used as the reporter strain, and *V. campbellii* ATCC BAA-1119 was used as the positive control. *Escherichia coli* DH5α was used as a negative control. *P. fragi* ATCC 4973^T^ and *P. aeruginosa* ATCC 27853^T^ were used as reference strains in this study. *E. coli*, *Vibrio*, and *Pseudomonas* strains were stored in 20% glycerol stocks prepared in LB broth, marine broth, and nutrient broth, respectively.

### 2.3. DNA Isolation, PCR, and Sequencing

Genomic DNA was isolated using the PureLink^TM^ Genomic DNA Mini Kit (Thermo Fisher Scientific Inc., Waltham, MA, USA). The *P. fragi*-related isolates were initially screened by PCR (T100^TM^ Thermal Cycler, Bio-Rad Laboratories, Hercules, CA, USA) using the primers carA-R and fraF, which target the carbamoyl phosphate synthase (*carA*) gene of *P. fragi*, in a species-specific PCR assay, as described by Ercolini et al. [26].

Molecular identification of the *P. fragi*-related isolates was performed based on their *rpo*D gene sequences. PCR amplification of the *rpoD* gene was carried out using Taq DNA Polymerase Master Mix (Ampliqon, Odense, Denmark) with primers PsEG30F (5′-ATYGAAATCGCCAARCG-3′) and PsEG790R (5′-CGGTTGATKTCCTTGA-3) [27]. The PCR conditions were as follows: an initial denaturation at 95 °C for 5 min; 30 cycles of denaturation at 95 °C for 30 s, annealing at 60 °C for 30 s, and extension at 72 °C for 30 s; followed by a final extension at 72 °C for 5 min. PCR products were purified using the PureLink^TM^ PCR Purification Kit (Thermo Fisher Scientific Inc., Waltham, MA, USA). After purification, the PCR products were sequenced by MedSantek (Istanbul, Türkiye). The *rpoD* gene sequences obtained from the isolates were deposited in the NCBI GenBank database under accession numbers PV974199–PV974291 (see Table S1 for details). Percent sequence identities to closely related taxa were determined using the BLASTn2.17 algorithm through the NCBI BLAST web interface (https://blast.ncbi.nlm.nih.gov/Blast.cgi, accessed on 16 June 2026) based on *rpoD* gene sequences (Table S1).

### 2.4. Phylogenetic Analysis

Phylogenetic analysis was performed using MEGA11 software [28], employing the Maximum Likelihood method based on the Tamura–Nei model. The final dataset included 635 positions from the *rpoD* sequences. The type strains used in the phylogenetic analysis and their corresponding accession numbers are as follows: *P. bubulae* TH39^T^ (NZ_UYJA01000004), *P. fragi* ATCC 4973^T^ (FN554466), *P. kulmbachensis* V3/3/4/13^T^ (NZ_CAVMKE010000009), *P. deceptionensis* M1^T^ (GU936596), *P. psychrophila* DSM 17535T (FN554506), *P. versut*a L10.10^T^ (KY264773), *P. paraversuta* V4/DAB/S4/2a^T^ (NZ_CAJFCL010000009), *P. saxonica* DSM 108989^T^ (MN068213), *P. lundensis* LMG 13517^T^ (FN554479), *P. weihenstephanensis* DSM 29166^T^ (KP738727), *P. taetrolens* IAM1653^T^ (AB039523), *P. endophytica* BSTT44^T^ (LN624763), *P. helleri* DSM 29165^T^ (KP738722), and *P. fluorescens* IAM12022^T^ (AB039545).

### 2.5. Motility Assays

For the swimming motility assay, tryptone medium containing 1% tryptone (Neogen, Lansing, MI, USA), 0.5% NaCl (Isolab™, Eschau, Germany), and 0.3% agar (Liofilchem^®^, Roseto degli Abruzzi, Italy) was prepared, and the isolates were inoculated into the medium using a sterile toothpick [29]. Following incubation at 25 °C for 16 h, the diameter of the swimming zone was measured with a ruler as an indicator of swimming motility. Swarming plates were prepared with 0.5% agar (Liofilchem^®^), 8 g/L nutrient broth, and 5 g/L dextrose (Daejung, Seogwipo, Republic of Korea). The isolates were inoculated at the center of the agar using a sterile toothpick and incubated at 25 °C for 24 h [30]. Swarming motility was assessed by measuring the diameter of the spreading zone. For the twitching motility assay, LB agar medium containing LB broth (Invitrogen, Carlsbad, CA, USA) and 1% agar was poured into Petri dishes [29,30]. The isolates were stab-inoculated into the agar using a sterile toothpick, ensuring contact with the bottom surface of the polystyrene Petri dishes, and incubated at 25 °C for 24 h. Following incubation, the agar was removed, and unattached cells were washed away. The adherent cells were then stained with 1% crystal violet, and twitching motility was quantified by measuring the diameter of the stained zone. For the calculation of the percentage of motile isolates, spreading zones < 5 mm were considered negative for motility.

### 2.6. Biofilm Production

An initial qualitative screening for biofilm formation was performed on Congo red agar (CRA) as previously described [31]. The CRA medium consisted of brain heart infusion broth (Sigma-Aldrich, St. Louis, MO, USA; 37 g/L), sucrose (Isolab; 50 g/L), agar (10 g/L), and Congo red (Isolab; 0.8 g/L). The isolates were streaked onto the agar and incubated at 25 °C for 48 h. Isolates producing black or dark red colonies were considered biofilm-positive.

The 35 CRA-positive meat-derived isolates, together with the two biofilm-forming reference strains, were further evaluated using the crystal violet microtiter plate assay [32,33]. Isolates grown overnight on nutrient agar were suspended in 0.85% physiological saline and adjusted to 0.5 McFarland using a densitometer (Den-1, Grant Instruments, Royston, UK). A 20 μL aliquot of each bacterial suspension was added to sterile 96-well polystyrene microtiter plates containing 180 μL of Tryptic Soy Broth (TSB, BD Bacto™, Franklin Lakes, NJ, USA). Sterile culture medium was used as the negative control. The plates were incubated at 4 °C and 25 °C for 1, 4, and 7 days. Following incubation, the culture medium was removed, and the wells were gently washed three times with sterile distilled water to remove non-adherent cells. The wells were then fixed with methanol (Isolab) for 15 min and stained with 1% (*w*/*v*) crystal violet (Carlo Erba Reagents, Cornaredo, Italy) for 5 min. After washing three times with distilled water and drying, the bound crystal violet was solubilized with 33% acetic acid (Merck), and absorbance was measured at 590 nm. Assays were performed in three independent experiments with four technical replicates per isolate.

### 2.7. AI-2 Activity Bioassay

*Vibrio campbellii* reporter strains, known for their bioluminescence production in response to AI-2, can be used to assay AI-2 activity. The bioassay was performed as described by Surette and Bassler [34]. An overnight culture of *V. campbellii* ATCC BAA-1117 (luxN::tn5Kan), which only responds to AI-2 molecules, was diluted 1:5000 in freshly prepared AB medium (17.5 g/L NaCl, 12.3 g/L MgSO4 (Sigma-Aldrich Co.), 2 g/L of casamino acids (Neogen), 10 mL/L of 1 M potassium phosphate (Sigma-Aldrich Co.) (pH 7), 10 mL/L of 0.1 M L-arginine (Maclin Group, Bury Saint Edmunds, UK), and 10 mL/L of glycerol; L-arginine was sterilized through a 0.22 µm filter (Sterile Syringe Filters, Isolab); other ingredients were autoclaved after preparation). All strains were adjusted to a 0.5 McFarland standard prior to incubation. Cell-free supernatants (CFS) were collected after a defined incubation period (6 h) by centrifugation at 5000 *g* for 10 min and subsequently sterilized using 0.22 µm filters [35]. The CFS of *V. campbellii* ATCC BAA-1119 was used as the positive control. Ten microliters of each CFS were mixed with 90 µL of the diluted reporter strain suspension in a 96-well microplate. *E. coli* DH5α was included as a biological negative control. In addition, a reporter-only control consisting of diluted *V. campbellii* ATCC BAA-1117 in assay medium without test CFS was included to determine baseline luminescence. Measurements were taken hourly using an INNO-M microplate reader (LTEK, Seongnam-si, Republic of Korea), and the assay was terminated at the onset of increased luminescence in the reporter-only control [34]. AI-2-like activity was considered detectable when cell-free supernatants induced reporter luminescence above the negative-control/background response at the selected reading point.

To prevent cross-well interference in 96-well plates, samples were arranged with empty wells left between them. This setup minimized false luminescence signals, as wells positioned adjacent to the positive control (*V. campbellii* ATCC BAA-1119) or highly luminescent samples tended to show artificially elevated readings. To further ensure accuracy, the positive control was measured on a separate plate. The assay was performed in three independent experiments with four technical replicates per isolate.

### 2.8. Statistical Analysis

Statistical analyses were conducted using Minitab (Version 22.1.0, Minitab, LLC, State College, PA, USA). Biofilm data were analyzed using a two-way repeated-measures ANOVA, with temperature (4 °C and 25 °C) and incubation time (days 1, 4, and 7) as within-isolate factors. Statistical significance was set at *p* < 0.05. Principal Component analysis (PCA) was also performed using the correlation matrix to identify patterns and correlations among the variables, with variables standardized to zero mean and unit variance prior to analysis to account for differences in measurement units and scales.

## 3. Results and Discussion

### 3.1. Molecular Identification of Pseudomonas Isolates

A total of 162 isolates were analyzed, of which 93 were identified as putative *P. fragi*-related isolates based on PCR screening using *P. fragi*-specific primers targeting the *carA* gene [26]. Molecular identification was then performed by sequencing the *rpoD* region, which provides higher taxonomic resolution than the *16S rRNA* gene in *Pseudomonas* [27,36,37].

**Figure 1:**
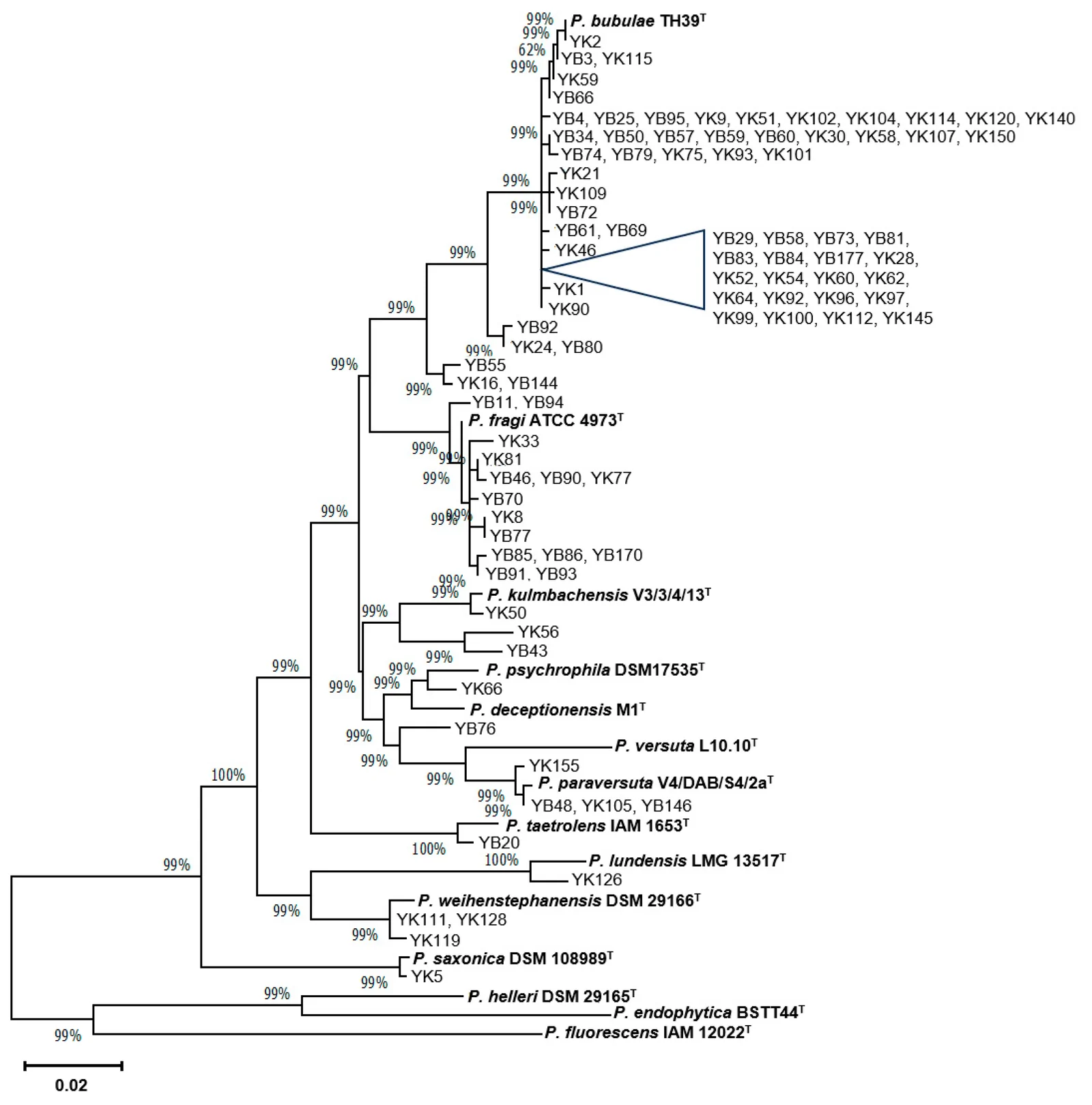
Phylogenetic tree of *Pseudomonas* isolates based on *rpoD* gene sequences, constructed using the maximum likelihood method. Numbers at the nodes indicate bootstrap support values; sequence identity values are provided in Supplementary Table S1. Isolates are labeled according to strain codes (YK and YB series).

The majority of the isolates (*n* = 57) were identified as *P. bubulae*, a recently described species closely related to *P. fragi* (Figure 1, Table S1), whose type strain was also isolated from bovine meat [38]. Additionally, *P. fragi* was the second-most frequently identified species (*n* = 15). Other isolates were assigned to *P. kulmbachensis*, *P. psychrophila, P. deceptionensis*, *P. paraversuta, P. taetrolens, P. lundensis*, *P. weihenstephanensis*, and *P. saxonica* (Figure 1, Table S1). Eight additional isolates were placed in distinct clades—six clustering near *P. bubulae* and two near *P. kulmbachensis*—which may indicate the presence of previously uncharacterized species.

Despite initial screening targeting *P. fragi*, sequencing results revealed the presence of multiple *Pseudomonas* species, as the PCR assay developed by Ercolini et al. [5] predates the formal description of many closely related species. For example, *P. weihenstephanensis* was described in 2016 [39], *P. bubulae* in 2019 [38], *P. saxonica* in 2020 [40], *P. paraversuta* in 2021 [41], and *P. kulmbachensis* as recently as 2024 [42]. As such, the assay was not designed to capture the full species diversity now recognized within the *P. fragi* complex. Similar molecular diversity among putative *P. fragi* isolates has also been observed in previous studies, including one that used RAPD-PCR and rep-PCR fingerprinting [5] and another based on whole-genome analysis [43].

The additional isolates that formed distinct clades on the phylogenetic tree may represent genetically distinct lineages within the *P. fragi*-related group. Two clades, each composed of three isolates (YK24, YB80, and YB92 and YB55, YK16, and YB144), clustered near *P. bubulae*, exhibiting *rpoD* percent sequence identities of approximately 98% and 96.5%, respectively—both below the >99% identity typically observed for confirmed *P. bubulae* strains (Table S1). Another group, consisting of isolates YK56 and YB43, showed 96–97% sequence identity to the *P. kulmbachensis* type strain (Table S1). These groups may represent previously unrecognized taxa and warrant further investigation through genome-based approaches such as average nucleotide identity (ANI) and digital DNA–DNA hybridization (dDDH).

**Figure 2:**
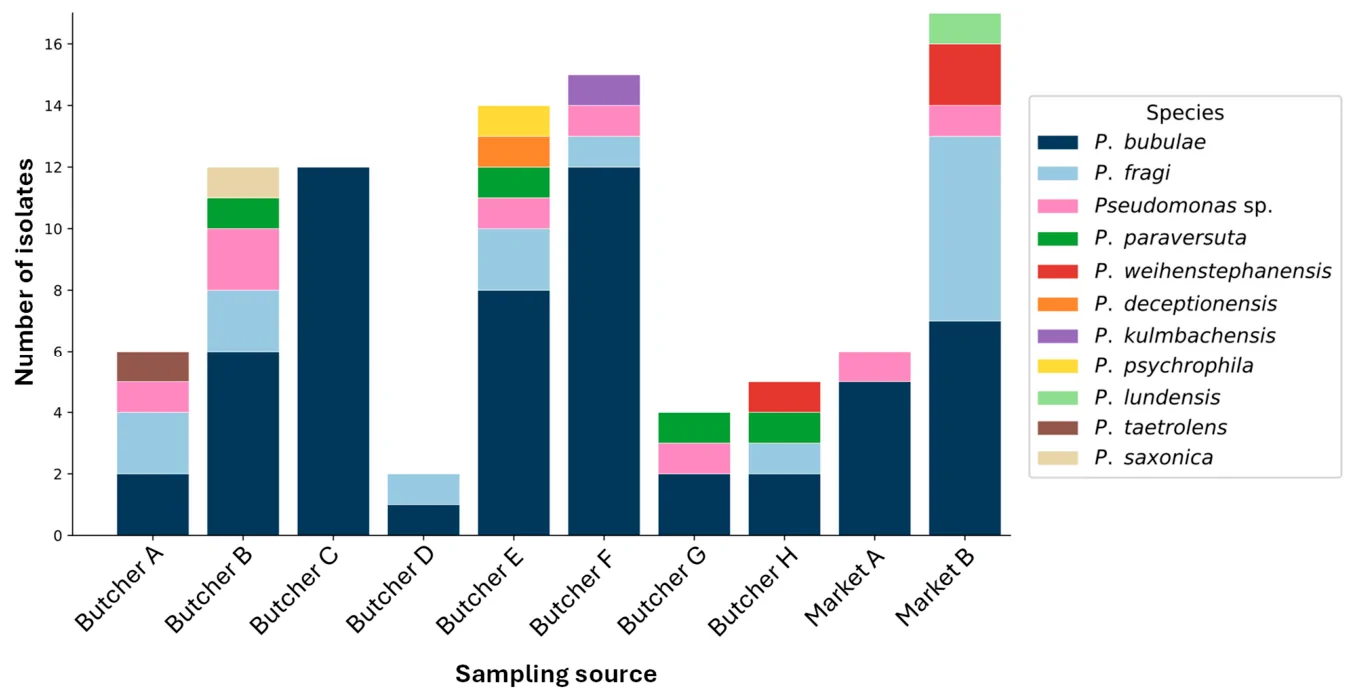
Distribution of identified *Pseudomonas* species among sampling sources.

Although *P. fragi* has traditionally been reported as the dominant species associated with aerobically stored refrigerated red meat [26,44,45], the overall isolate diversity observed in this study, along with the high frequency of *P. bubulae*, suggests that *P. bubulae*—the recently recognized member of the *P. fragi* group— may be more prevalent among *P. fragi*-related isolates associated with this niche than previously recognized. Importantly, *P. bubulae* was consistently detected and was the predominant species across all sampling sources (Figure 2), supporting its widespread distribution among the examined *P. fragi*-related isolates from steak and minced meat samples. These findings suggest that *P. fragi*-related isolates associated with meat may be more diverse than previously assumed, particularly in light of recent taxonomic updates in the *Pseudomonas* genus. Based on the molecular identification and phylogenetic analysis, 81 of the 93 isolates initially identified as putative *P. fragi*-related isolates were selected for subsequent analyses, as they clustered most closely with *P. fragi* and its closely related species, *P. bubulae* and *P. kulmbachensis*.

### 3.2. Motility Phenotypes of Pseudomonas Isolates

Bacterial motility facilitates colonization of nutrient-rich environments and may contribute to surface attachment and subsequent biofilm development [46]. Among the 81 isolates, swimming and twitching were the most prevalent phenotypes, detected in 77 (95.1%) and 78 (96.3%) isolates, respectively, while swarming was observed in 42 isolates (51.9%) (Table S2). Motility extent varied markedly, with swimming zones ranging from 6 to 35 mm, swarming from 4 to 25 mm, and twitching from 7 to 81 mm. Strain-level heterogeneity was particularly evident for isolate YB43, which exhibited the largest twitching zone (81 mm) together with the highest swarming diameter (25 mm). Similar variability has been reported for dairy-associated *Pseudomonas* spp. [47], with swimming, swarming, and twitching frequencies of approximately 46%, 34%, and 26%, respectively. Notably, meat-associated isolates in this study exhibited higher frequencies of swimming and twitching. However, comparisons of migration diameters across studies should be interpreted cautiously, as agar concentration, medium composition and strain origin can substantially influence the motility readouts [48].

### 3.3. Biofilm Formation Capacity

**Figure 3:**
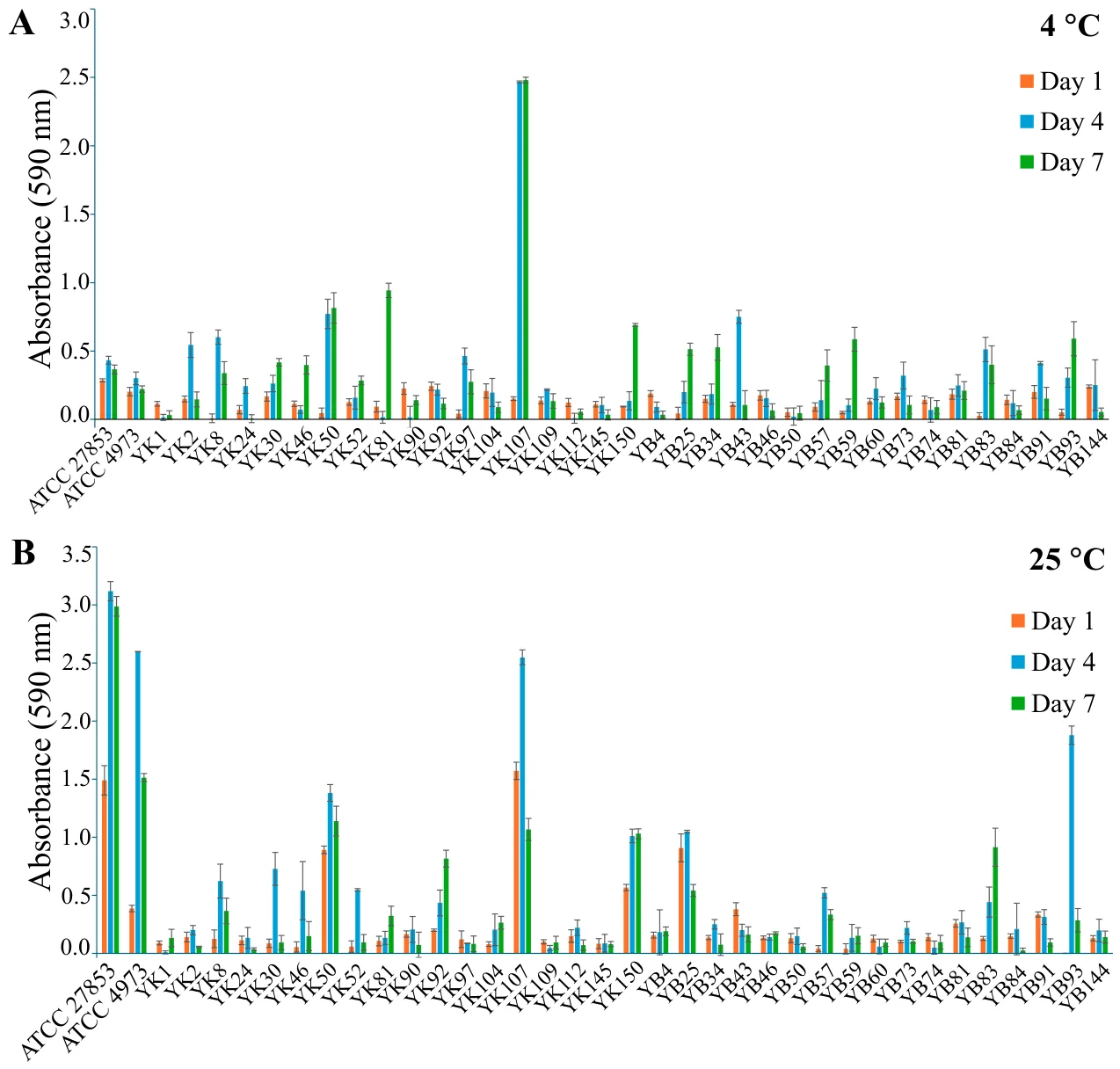
Biofilm production of Congo red-positive *Pseudomonas* spp. at 4 °C (**A**) and 25 °C (**B**) during a 7-day incubation period. ATCC 27853 (*Pseudomonas aeruginosa*) and ATCC 4973^T^ (*Pseudomonas fragi*) were used as reference strains. YK and YB denote the studied isolates.

The qualitative CRA assay revealed that 35 out of 81 meat-derived isolates were CRA-positive based on black or dark-red colony formation, as were the biofilm-forming reference strains *P. aeruginosa* ATCC 27853 and *P. fragi* ATCC 4973^T^ [49,50]. CRA-positive isolates were considered presumptive biofilm formers [51], and biofilm formation was subsequently quantified using the crystal violet microtiter plate assay (Figure 3). OD_590_ measurements were used to quantitatively compare biofilm biomass among isolates.

Most isolates, as well as the reference strains, produced higher levels of biofilm at 25 °C than at 4 °C (Figure 3). Nevertheless, several isolates maintained comparable biofilm production across both temperatures, while a few exhibited higher biofilm levels at 4 °C. These findings indicate substantial strain-to-strain variability in the temperature-dependent regulation of biofilm formation.

Such variability is consistent with previous reports showing that *Pseudomonas* spp. can maintain biofilm formation across a broad temperature range, including chilled conditions typical of meat-processing environments [52]. Indeed, the ability of *Pseudomonas* spp. to survive and remain metabolically active at low temperatures has been widely reported and is linked to their metabolic versatility [53]. Several studies have demonstrated that biofilm formation persists under refrigeration conditions, where pseudomonads may establish and maintain biofilms at ~4 °C [54,55]. For example, *P. fluorescens*, *P. lundensis*, and *P. psychrophila* isolates have been reported to form biofilms at refrigeration temperatures comparable to or even higher than those at room temperature [8,56]. Together with our findings, these studies suggest that certain meat-associated *Pseudomonas* spp. can maintain or enhance biofilm-forming capacity under chilled conditions, which may contribute to their persistence in refrigerated food-processing environments.

To evaluate the overall effects of temperature and incubation time on biofilm formation, OD_590_ data from the 37 strains included in the quantitative biofilm assay were analyzed using a two-way repeated-measures ANOVA. Significant main effects were detected for temperature (F(1, 36) = 4.199, *p* = 0.048) and incubation time (F(2, 72) = 11.660, *p* < 0.001), whereas the temperature × incubation time interaction was not significant (F(2, 72) = 2.258, *p* = 0.112). Overall, mean biofilm biomass was higher at 25 °C than at 4 °C. Descriptively, at 4 °C, mean biofilm biomass increased from day 1 to day 4 and remained at a comparable level on day 7, whereas at 25 °C, mean biofilm biomass increased from day 1 to day 4 and subsequently decreased by day 7. Thus, although the temperature × incubation time interaction was not statistically significant, the descriptive profiles indicated that biofilm biomass was maintained during the later incubation period under chilled conditions. These observations are broadly consistent with established models of biofilm development, in which biomass may increase following initial attachment and subsequently stabilize or decrease during later stages of development [4,53].

Similar to our findings, Wickramasinghe et al. [4] demonstrated that biofilm mass produced by *P. fragi* increased from day 3 to 7 during storage at 4 °C, while at 25 °C a decrease in cell density and biofilm disintegration was observed. Liu et al. [8] similarly reported that *P. fluorescens* and *P. psychrophila* isolates from spoiled beef and fish exhibited the highest biofilm production at 4 °C after day 3, with a decline or stabilization over prolonged incubation. These patterns may reflect the longer time required for biofilm development at lower temperatures and, in some strains, delayed but sustained biomass accumulation under chilled conditions [55]. These observations suggest that temperature-dependent differences may reflect stage-specific patterns of biofilm development, with some meat-associated pseudomonads capable of sustaining or even increasing biofilm biomass at low temperatures over time.

Although the crystal violet assay provides a useful approach for comparative quantification of biofilm biomass across a large number of isolates, it does not provide information on biofilm architecture, thickness, or spatial organization. Future microscopy-based studies targeting selected high-biofilm-producing strains could therefore provide a more detailed characterization of biofilm structure and organization.

### 3.4. Autoinducer-2 (AI-2) Activity

**Figure 4:**
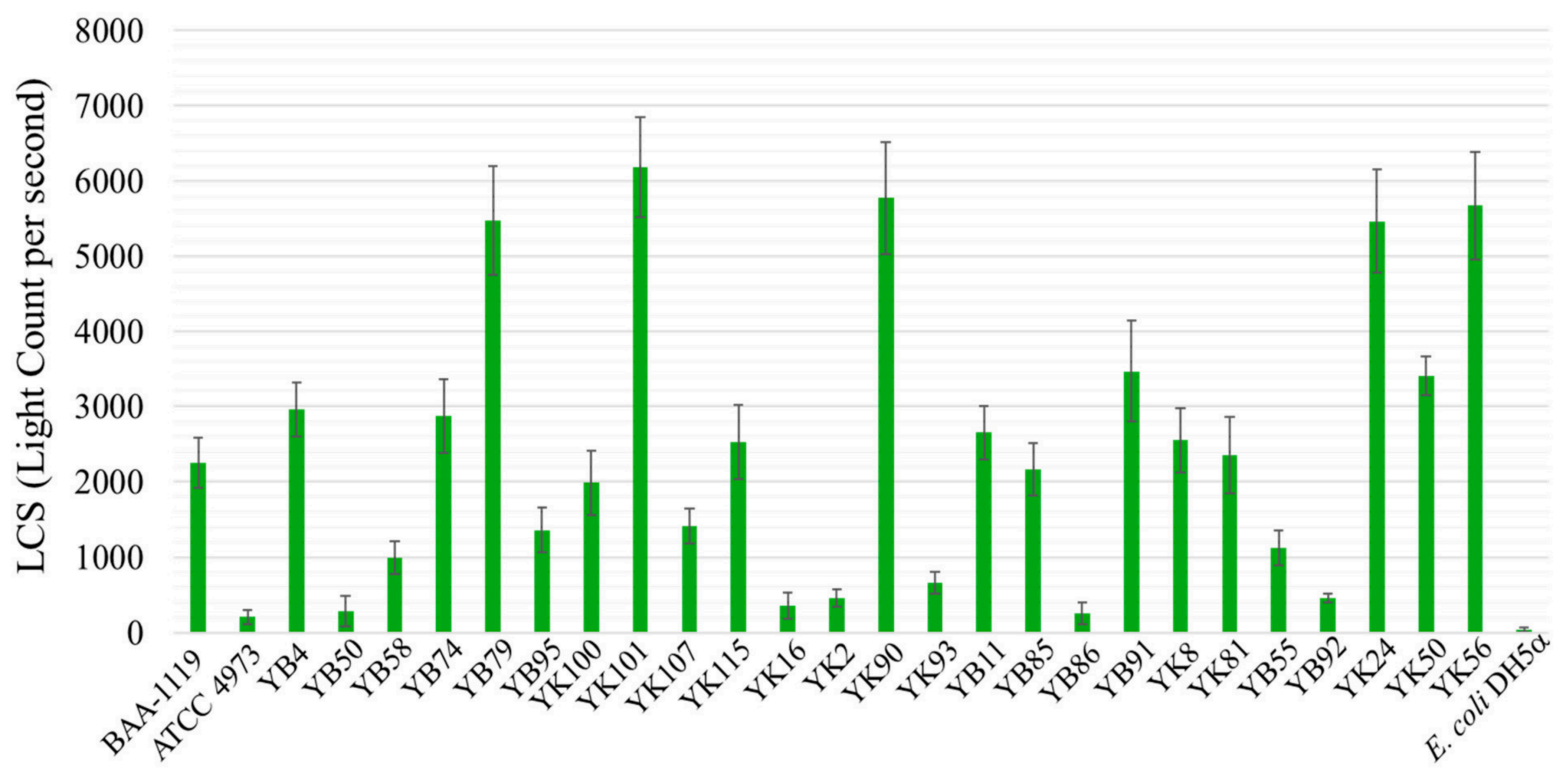
AI-2 activity of isolates measured as luminescence (light count per second, LCS). *Vibrio campbellii* ATCC BAA-1119 was used as a positive control, *Pseudomonas fragi* ATCC 4973^T^ as a reference strain, and *Escherichia coli* DH5α as a negative control. YK and YB denote isolate codes.

In this study, out of 81 isolates, 25 exhibited AI-2 activity (Figure 4). The highest AI-2 activity was observed in strains YK101, YK90, YK24, YK56 and YB79. *P. fragi* ATCC 4973^T^ showed lower AI-2 activity compared to the tested isolates. Marked variability in luminescence intensity was observed among isolates, with several strains (e.g., YB4, YK115, YB11, YB74, YK8, and YK81) exhibiting activity comparable to the positive control, whereas others showed relatively low levels. This variability suggests strain-dependent differences in AI-2-mediated signaling among meat-associated pseudomonads.

Previous studies have shown that AI-2 activity has been detected in various food matrices, including fish, poultry, and meat products [57,58]. However, reports on AI-2 activity in *Pseudomonas* spp. isolated from meat remain limited, with only a few studies describing its occurrence [25]. In this context, our findings provide additional evidence for AI-2 activity in meat-associated *Pseudomonas* isolates, highlighting their potential role in microbial communication and spoilage processes in retail beef.

Beyond meat systems, AI-2-mediated QS has been implicated in microbial interactions and biofilm formation in diverse food environments. For instance, Lu et al. [59] reported that AI-2 activity on Roma tomatoes promoted *E. coli* biofilm formation. Similarly, Silagyi et al. [60] observed AI-2 activity in *E. coli* O157:H7 in various food matrices, with the highest levels detected in spinach. In fermented foods, lactic acid bacteria are also recognized as strong AI-2 producers [61,62].

Collectively, these studies emphasize the potential ecological relevance of AI-2-mediated signaling in food ecosystems and its possible role in microbial growth, biofilm formation, and spoilage. Although most QS research has focused on pathogenic bacteria [63,64], these concepts are increasingly being applied to food microbiology. However, further studies are required to elucidate the QS-mediated interactions in *Pseudomonas* spp. in raw meat environments. Notably, this study, together with Ferrocino et al. [25], represents one of the few investigations assessing AI-2 activity in meat-associated *Pseudomonas* spp. and its association with biofilm formation.

An additional consideration is that AI-2 activity detected in *Pseudomonas* spp. may not necessarily reflect a canonical LuxS-dependent QS system. In the classical pathway, LuxS converts S-ribosylhomocysteine into 4,5-hihydroxy-2,3-pentandione, the precursor of AI-2 [65,66]. However, several *Pseudomonas* species, including *P. aeruginosa*, lack a recognizable *luxS* homolog and are therefore not expected to synthesize AI-2 via this pathway, although they can respond to exogenous AI-2 signals [67,68]. For example, *P. aeruginosa* detects AI-2 via the chemoreceptors PctA and TlpQ, enhancing chemotaxis and biofilm formation [69].

Interestingly, Ferrocino et al. (2009) [25] reported detectable AI-2-like activity in meat-derived *P. fragi* isolates despite the absence of a canonical *luxS* gene in *Pseudomonas* spp. Consistent with previous reports [67,68,69], our analysis of available genome sequences of representative meat-associated *Pseudomonas* species likewise failed to identify a recognizable *luxS* homolog. Furthermore, AI-2-like molecules may also arise through LuxS-independent routes, including spontaneous conversion of ribulose-5-phosphate, an intermediate of the pentose phosphate pathway [70]. Therefore, AI-2 activity detected in *Pseudomonas* spp. may reflect strain-specific differences in AI-2 activity and/or sensing mechanisms rather than a single conserved LuxS-dependent regulatory pathway.

### 3.5. Correlation of Motility, Biofilm Formation, and AI-2 Activity Among Pseudomonas Isolates

**Figure 5:**
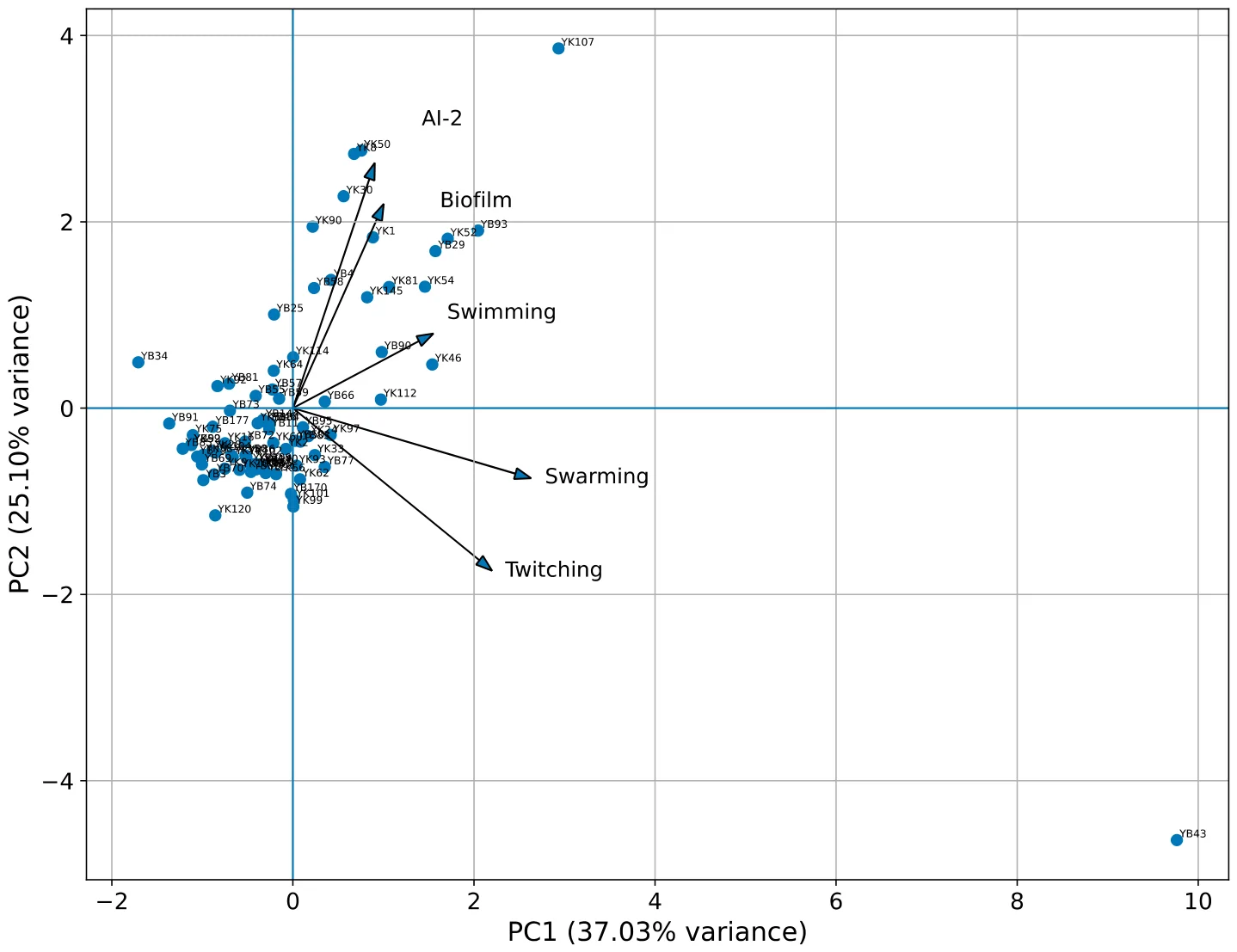
Principal component analysis (PCA) of 81 isolates, including *Pseudomonas fragi* ATCC 4973^T^, based on standardized autoinducer-2 (AI-2) activity (LCS), biofilm formation (OD_590_), and motility traits (twitching, swimming, and swarming; mm). Arrows indicate variable loadings on principal components. YK and YB denote isolate codes.

In this study, principal component analysis (PCA) was also performed on 81 meat-derived isolates to explore patterns and relationships among AI-2 activity (LCS), biofilm formation (OD_590_), and motility traits (twitching, swimming, and swarming; mm) (Figure 5). For the PCA, quantitative OD_590_ values obtained from the 35 CRA-positive isolates were used as the biofilm variable, while CRA-negative isolates were coded as zero for this variable. The first two principal components accounted for 62.12% of the total variance, with PC1 explaining 37.03% and PC2 explaining 25.10%. PC1 was primarily associated with motility-related traits, particularly twitching and swarming, whereas PC2 was mainly driven by AI-2 activity and biofilm formation. The similar direction of the AI-2-like activity and biofilm loadings on PC2 indicates that these traits contributed similarly to this component within the multivariate phenotypic space (Figure 5).

The PCA score plot revealed a heterogeneous distribution of isolates, without clearly defined clusters. Most isolates were positioned near the center of the ordination space, indicating relatively limited contributions to either principal component. In contrast, several isolates showed distinct separation along the principal components. Notably, isolate YK107 was strongly separated along PC2, consistent with its high biofilm formation, while YB43 was positioned toward the positive side of PC1, reflecting pronounced motility-related traits. Isolate YB93 also showed displacement along PC2, corresponding to elevated biofilm formation despite the absence of detectable AI-2 activity.

To directly examine the relationship between AI-2-like activity and quantitatively measured biofilm formation, Spearman’s rank correlation was additionally performed for the 35 isolates subjected to the quantitative biofilm assay. No significant monotonic correlation was detected between AI-2-like activity and biofilm formation (Spearman’s ρ = 0.057, 95% CI: −0.282 to 0.383, *p* = 0.744).

Although AI-2-like activity and biofilm formation showed similar loading directions on PC2, the direct Spearman correlation analysis did not demonstrate a significant monotonic relationship between these two traits. Therefore, the PCA pattern should be interpreted as an exploratory multivariate co-loading rather than evidence of a direct or statistically significant association between AI-2-like activity and biofilm formation. The considerable strain-to-strain variability observed in the present study further indicates that biofilm formation is unlikely to be explained by AI-2-like activity alone and may involve additional regulatory and physiological mechanisms. Further molecular and transcriptomic studies are required to elucidate the regulatory basis of these phenotypic traits.

The relationship between AI-2 activity and biofilm formation has been investigated in various microbial systems, with studies reporting associations between AI-2 and biofilm-related phenotypes. For instance, Zhang et al. [71] reported that the addition of synthetic AI-2 enhanced *Fructilactobacillus sanfranciscensis* (formerly *Lactobacillus sanfranciscensis*) growth and biofilm production, while Jiang et al. [72] showed that deletion of *luxS* in a shellfish-derived *Vibrio parahaemolyticus* strain reduced AI-2 activity, motility, and biofilm-forming capacity. Collectively, these studies indicate that AI-2-mediated signaling may play a role in modulating biofilm-related phenotypes.

However, not all studies have reported a significant association between AI-2 activity and biofilm production, particularly in food-associated environments. For example, Van Houdt et al. [73] found no direct correlation between biofilm-forming ability and the production of AHL or AI-2 signaling molecules in Gram-negative bacteria isolated from food-processing environments, while Yoon & Sofos [74] reported that AI-2 activity did not substantially influence biofilm formation by *Salmonella* and *E. coli* O157 on food contact surfaces. More recently, Gu et al. [75] found that exogenous synthetic AI-2 had no significant effect on biofilm formation in *Enterococcus faecium* and *Limosilactobacillus fermentum* (formerly *Lactobacillus fermentum*), suggesting that the effects of AI-2 on biofilm formation may be species- or context-dependent. Consistent with these observations, the present study identified isolates exhibiting high biofilm-forming capacity despite low or undetectable AI-2 activity; therefore, factors other than AI-2 activity may contribute to biofilm development in meat-associated *Pseudomonas* spp.

Taken together, these findings highlight the complexity of QS-associated phenotypes in spoilage bacteria and emphasize the value of multivariate approaches for exploring phenotypic diversity that may not be evident through univariate analyses. The lack of a significant direct relationship between AI-2 activity and biofilm formation in the present study further suggests that additional studies are needed to better understand the regulatory mechanisms underlying these traits and microbial interactions among spoilage-associated pseudomonads.

AI-2 quantification should also be interpreted with caution, since signal production and accumulation can be influenced by growth conditions, medium composition, and other environmental factors [34,35]. Therefore, the AI-2 activity reported here likely reflects the physiological state of the isolates under the experimental conditions rather than their intrinsic signaling capacity. Future studies employing complementary analytical approaches, such as HPLC and particularly LC–MS/MS, could enable more accurate quantification and chemical characterization of AI-2-related compounds, allow monitoring of their temporal changes during bacterial growth, and help assess the specificity of the reporter response. Nevertheless, the present study provides an initial phenotypic overview of meat-associated *Pseudomonas* isolates by integrating taxonomic diversity with biofilm formation, motility, and AI-2 activity, thereby establishing a basis for future mechanistic studies on signaling and spoilage-associated phenotypes.

From a food safety perspective, the relevance of meat-derived *Pseudomonas* may extend beyond their established role in spoilage. Although the isolates examined in the present study were investigated primarily as spoilage-associated bacteria and their pathogenic potential was not assessed, biofilm-forming pseudomonads may influence the persistence of foodborne pathogens within multispecies communities [76]. Under simulated chilled-beef processing conditions, dual-species biofilms of *P. fluorescens* and *Listeria monocytogenes* resulted in increased *L. monocytogenes* populations at 4 °C, suggesting that pseudomonad-associated biofilms may provide favorable conditions for pathogen persistence [77]. Similarly, high-biofilm-forming *Pseudomonas* strains, including *P. fragi*, have been shown to contribute to the persistence of *Salmonella enteritidis* in dual-species biofilms on slaughterhouse surfaces [78]. Interspecies chemical communication may further influence such interactions, as signals produced by food-associated *Pseudomonas* have been shown to modulate the motility and biofilm formation of *L. monocytogenes* [76]. Thus, the biofilm-forming capacity and AI-2-like activity observed in some isolates may have indirect food safety relevance, particularly in the context of multispecies biofilms and microbial interactions, although their potential roles in pathogen persistence require direct investigation.

## 4. Conclusions

In conclusion, this study revealed substantial phenotypic diversity among meat-associated *Pseudomonas* isolates. Among the *P. fragi*-related isolates examined, *P. bubulae* was the predominant species, highlighting the value of updated taxonomic approaches for resolving species identities within this group. Biofilm formation was influenced by temperature and was generally lower at 4 °C during the early stages of incubation, although several strains maintained substantial biofilm-forming capacity under chilled conditions. In addition, AI-2 activity was detected in a subset of isolates, and multivariate analysis showed that AI-2 activity and biofilm formation contributed to a similar phenotypic dimension, whereas motility traits represented a largely independent phenotypic dimension. However, direct correlation analysis did not reveal a significant monotonic relationship between AI-2 activity and biofilm formation, indicating that biofilm formation is likely influenced by multiple regulatory and physiological factors. Collectively, these findings provide a phenotypic framework for understanding the diversity of meat-associated *Pseudomonas* and identify traits and strain-level patterns that warrant further investigation. The strong biofilm-forming capacity and AI-2-like activity observed in some isolates may have broader food safety relevance, particularly in the context of microbial interactions and multispecies biofilms, warranting further investigation of their potential roles in the persistence of foodborne pathogens in meat-associated environments.

## Data Availability

Data are available from the corresponding author upon reasonable request. The *rpoD* gene sequences have been deposited in the NCBI GenBank database under accession numbers PV974199–PV974291.

## Supplementary Materials

The following supporting information can be downloaded at https://www.mdpi.com/article/10.3390/foods15173044/s1. Table S1: Isolation sources and *rpoD* gene-based taxonomic identification of *Pseudomonas* isolates; Table S2: Motility characteristics of meat-associated *Pseudomonas* isolates.
